# Massive iliopsoas haematoma during the course of COVID‐19 pneumonia

**DOI:** 10.1002/rcr2.1070

**Published:** 2022-12-08

**Authors:** Hiroaki Nagano, Sara Takaesu

**Affiliations:** ^1^ Department of Respiratory Medicine Okinawa Chubu Hospital Uruma‐shi Japan

**Keywords:** bleeding, COVID‐19, haemorrhagic complication, heparin, iliopsoas haematoma

## Abstract

This report presents a case of life‐threatening iliopsoas haematoma in an immunosuppressed 86‐year‐old man with a history of prostate cancer during the clinical course of coronavirus disease 2019 (COVID‐19). The patient was hospitalized for COVID‐19‐associated pneumonia. One week after admission, he complained of pain in his right thigh when he changed his position. Laboratory findings revealed markedly progressive anaemia and elevated creatine phosphokinase levels. Chest computed tomography revealed a massive haematoma in the right iliopsoas muscle spreading to the retroperitoneal space. Considering the advanced age and status of the patient, he was treated with red blood cell transfusions and bed rest. Fortunately, the anaemia was improved, and the haematoma gradually reduced in size. It should be noted that even during isolation, careful physical examination is important. In addition, physicians who administer heparin to patients with COVID‐19, even if prophylactic, should be aware of bleeding complications.

## Graphical abstract

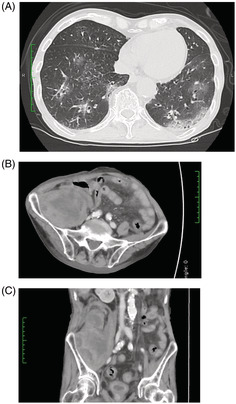

## INTRODUCTION

With increasing awareness of coronavirus disease 2019 (COVID‐19)‐associated coagulopathy, it has become clear that thrombotic symptoms are associated with prognosis.^1^ A recent multicentre study reported a rate of thrombotic complications as high as 9.5% despite standard anticoagulation prophylaxis, highlighting the critical importance of anticoagulation in the treatment of COVID‐19.^2^ Moreover, haemorrhagic events among patients with COVID‐19 have been recognized (overall rate 4.8%, fatal major bleeding 2.3%).^2^

We describe a case of a massive iliopsoas haematoma (IPH) developed during the treatment of COVID‐19.

## CASE REPORT

An 86‐year‐old man was referred to the emergency department with the chief complaint of difficulty in moving due to fever and general malaise. The patient had a history of pulmonary tuberculosis 40 years previously and smoking. He was taking abiraterone acetate for the treatment of prostate cancer and 5 mg prednisolone daily to prevent adrenal insufficiency. His fever had started 11 days before the visit and gradually worsened with a decrease in appetite and fatigue. The day before admission, the patient was found collapsed and immobile on the floor and was admitted to a hospital.

**FIGURE 1:**
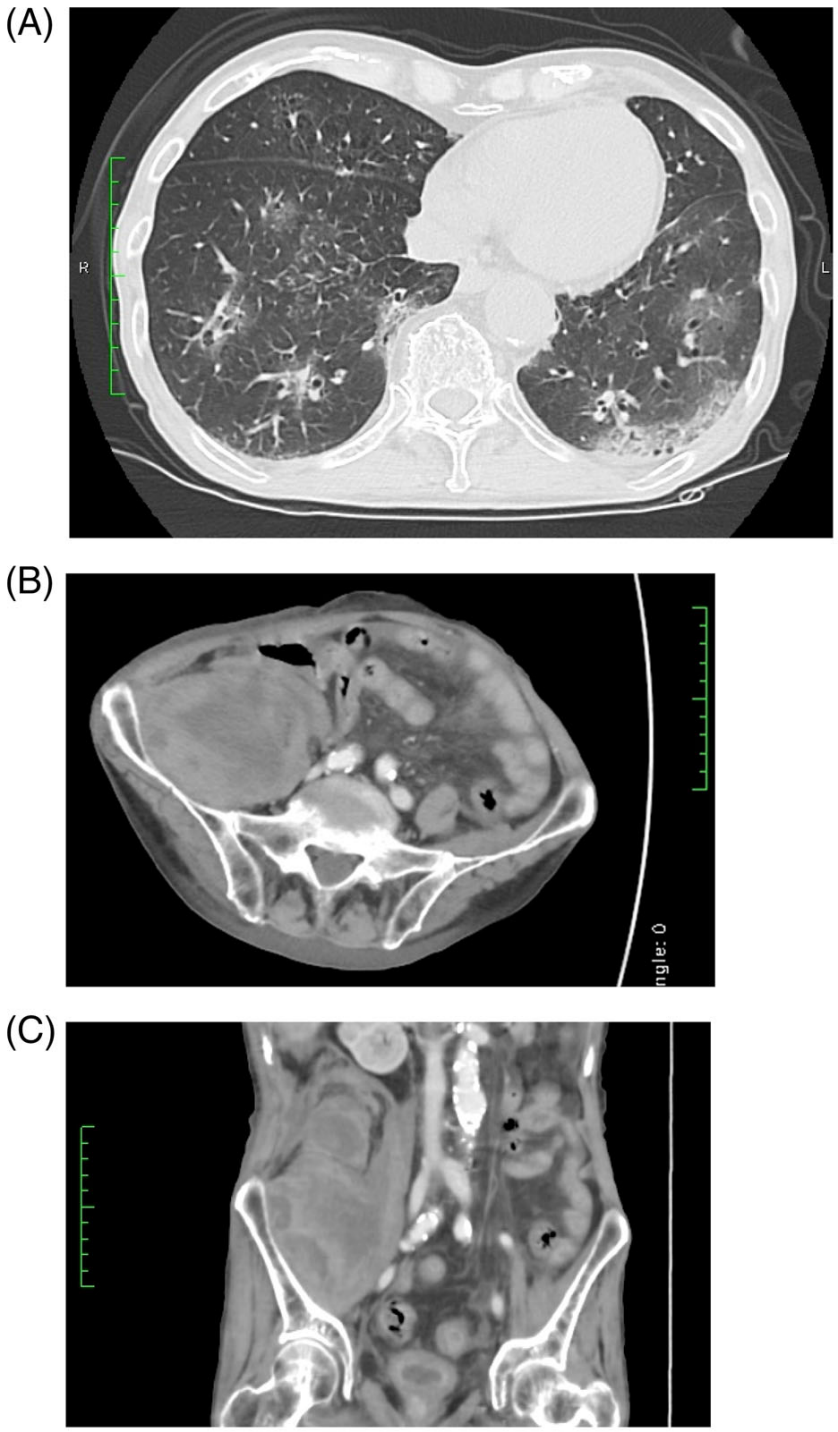
(A) Chest high‐resolution computed tomography revealing bilateral and multifocal ground‐glass opacities in predominately peripheral distribution. (B and C) Contrast‐enhanced abdominal and pelvic computed tomography showing a massive haematoma (6.0 × 9.5 × 10.5 cm) in the right iliopsoas muscle spreading to the retroperitoneal space

On admission, he was febrile, with a body temperature of 37.8°C, blood pressure of 120/60 mmHg, heart rate of 94 beats/min, and respiratory rate of 24 breaths/min. Although his oxygen saturation was 97% in ambient air, he seemed to breathe with difficulty. Chest computed tomography (CT) on admission showed patchy ground‐glass opacities in the bilateral lungs (Figure 1A). Laboratory findings showed normal complete blood count profile but elevated levels of C‐reactive protein at 9.91 and ferritin at 513.0 (normal, 50–200) ng/ml. Regarding the coagulation system, D‐dimer level was mildly elevated at 1.8 (cut‐off <1.0) μg/ml, but other data were within normal limits. The patient tested positive for COVID‐19 according to polymerase chain reaction for severe acute respiratory syndrome‐coronavirus‐2 performed using a nasal swab.

The patient received remdesivir (200 mg loading dose on day 1, followed by 100 mg daily for 4 days) and a subcutaneous injection of 5000 units of unfractionated heparin twice daily for prophylaxis of deep vein thrombosis.

One week after admission, the patient complained of pain in his right thigh when he changed his position. The attending physician carefully repeated the physical examination and detected psoas muscle signs. The patient's haemoglobin level dropped from 13.8 to 4.5 g/dl. The patient's coagulation system status was as follows: prothrombin time‐international normalized ratio was 1.47, activated partial thromboplastin time was 47.4 s, and D‐dimer level was slightly elevated at 1.4 ng/ml.

Contrast‐enhanced abdominal and pelvic CT revealed a massive haematoma (6.0 × 9.5 × 10.5 cm) in the right iliopsoas muscle spreading to the retroperitoneal space (Figure 1B, C). He immediately received 4 units of red blood cell transfusions. Although the radiologist considered interventional radiology to stop the bleeding, the attending physicians decided not to perform endovascular treatment considering the deterioration of respiratory condition due to pulmonary oedema caused by the transfusion.

Fortunately, the patient's vital signs were stable, and his haemoglobin level had stopped decreasing. One month later, CT showed that the haematoma had reduced in size. He started rehabilitation with a physiotherapist and was transferred to a rehabilitation hospital 2 months after haematoma onset. Throughout his hospitalization, prophylactic heparin was not readministered after haematoma development.

## DISCUSSION

IPH is a retroperitoneal haemorrhage involving the iliopsoas muscle, an important and often fatal complication of inpatient anticoagulation therapy.^2^ The psoas sign is a useful finding on physical examination.

COVID‐19 is associated with coagulopathy, including venous thromboembolism (VTE). Previous studies have reported VTE in 4.4% of hospitalized patients, with a high frequency ranging from 8.3% to 31% in critically ill patients.^3^, ^4^ Therefore, VTE prophylaxis in hospitalized patients with COVID‐19 is important. In contrast, there are several reports of IPH during COVID‐19 treatment. Recently, Vergori et al. reported seven cases of IPH in COVID‐19 inpatients, with a prevalence of 7.6 cases per 1000 admissions, which was approximately twice the previously reported incidence of IPH in intensive care units.^5^ In Japan, unfractionated heparin is commonly used due to insurance‐related factors, and several cases of IPH in patients receiving unfractionated heparin have been reported.^6^

IPH in patients with COVID‐19 may be caused by the increased coagulation function associated with COVID‐19 and the resulting haemorrhagic diathesis. Boira et al. reported that the haemorrhagic risk increases 10–14 days from disease onset in patients with COVID‐19, which is consistent with the clinical course of our patient.^7^

It is unclear whether a direct impact on vascular endothelial cells causes bleeding in COVID‐19. It has been hypothesized that severe acute respiratory syndrome coronavirus 2 has an affinity for angiotensin‐converting enzyme 2 receptors on vascular endothelial cells, which may cause vessel damage and lead to vessel wall rupture, and that dysfunction of the renin‐angiotensin‐aldosterone system may cause haemorrhagic complications.^8^

Although it is unknown whether bleeding events associated with COVID‐19 occur predominantly in the iliopsoas muscle, the likelihood of bleeding may be high at sites where angiotensin‐converting enzyme 2 receptors are abundantly expressed.^8^, ^9^

When treating COVID‐19 patients receiving prophylactic anticoagulation therapy, special attention should be paid to any symptoms suggestive of IPH, such as hip pain, femoral nerve palsy, and psoas muscle signs. In this case, the attending physician carefully repeated the physical examination and detected psoas muscle signs, leading to early CT imaging and diagnosis.

Here, we described a case of life‐threatening IPH during the clinical course of COVID‐19. Physicians who treat COVID‐19 patients with heparin, even if prophylactic, should be aware of bleeding complications. Thus, even during isolation, careful physical examination is important.

## AUTHOR CONTRIBUTIONS

Hiroaki Nagano is the first author and the corresponding author of this manuscript. Hiroaki Nagano drafted the manuscript and approved the final manuscript.

## CONFLICT OF INTEREST

None declared.

## ETHICS STATEMENT

The authors declare that appropriate written informed consent was obtained for the publication of this manuscript and accompanying images.

## Data Availability

Data sharing not applicable to this article as no datasets were generated or analysed during the current study.
